# Improving The Measurement Of Structural Racism To Achieve Antiracist Health Policy

**DOI:** 10.1377/hlthaff.2021.01489

**Published:** 2022-02

**Authors:** Rachel R. Hardeman, Patricia A. Homan, Tongtan Chantarat, Brigette A. Davis, Tyson H. Brown

**Affiliations:** University of Minnesota, Minneapolis, Minnesota.; Florida State University, Tallahassee, Florida.; University of Minnesota.; University of California San Francisco, San Francisco, California.; Duke University, Durham, North Carolina.

## Abstract

Antiracist health policy research requires methodological innovation that creates equity-centered and antiracist solutions to health inequities by centering the complexities and insidiousness of structural racism. The development of effective health policy and health equity interventions requires sound empirical characterization of the nature of structural racism and its impact on public health. However, there is a disconnect between the conceptualization and measurement of structural racism in the public health literature. Given that structural racism is a system of interconnected institutions that operates with a set of racialized rules that maintain White supremacy, how can anyone accurately measure its insidiousness? This article highlights methodological approaches that will move the field forward in its ability to validly measure structural racism for the purposes of achieving health equity. We identify three key areas that require scholarly attention to advance antiracist health policy research: historical context, geographical context, and theory-based novel quantitative and qualitative methods that capture the multifaceted and systemic properties of structural racism as well as other systems of oppression.

More than thirty years ago James Baldwin, one of the most notable voices on civil rights, reflected on the so-called progress of addressing racism in the US with the quote: “What is it you wanted me to reconcile myself to?… You always told me ‘It takes time.’ It’s taken my father’s time, my mother’s time, my uncle’s time, my brothers’ and my sisters’ time. How much time do you want for your progress?”^1^

Quite simply, time has run out. Progress toward racial equity has been elusive for more than 400 years, and the world is now in a moment that requires that it invest in a different way of doing things. In 2020 the world watched a Black man, George Floyd, Jr., be brutally murdered beneath the knee of a White police officer in Minnesota, and in the following year 229 more Black people lost their lives at the hands of police in the US.^2^ Throughout 2020 and 2021 Americans also watched a global pandemic disproportionately ravage Black communities across the country.^3^

Black communities are bearing the physical burdens of centuries of injustice, toxic exposures, and White supremacist violence. These burdens are wearing and tearing at bodies and cutting some lives short while preventing others from even beginning.^4,5^ At the root of this tragic reality is a legacy of structural racism.^4^ *Structural racism* refers to the “totality of ways in which societies foster racial discrimination through mutually reinforcing systems of housing, education, employment, earnings, benefits, credit, media, health care, and criminal justice. These patterns and practices in turn reinforce discriminatory beliefs, values, and the distribution of resources.”^5^ For example, prior research suggests that the legacies of structural racism—manifested in various historical and contemporary forms discussed in this article—create circumstances in which Black babies are too often born too soon and too small and don’t make it to their first birthday.^6,7^

Growing awareness of structural racism within (and outside of) public health discourse has led to calls for public health researchers, governmental public health practitioners, medical care providers, and policy makers to explicitly identify structural racism as a root cause of racial health inequity.^5,8,9^ Yet empirical research has been slow to quantify structural racism and its impact on public health.^10^ What isn’t measured cannot be managed, nor can it be valued.

During the late nineteenth and early twentieth centuries, Black-White disparities in mortality and morbidity were erroneously attributed to notions of biological racial inferiority.^11^ W. E. B. Du Bois, one of the first people to challenge this predominant notion, pushed for the systematic empirical investigation of social factors contributing to racialized health risk and health inequities.^12,13^ More than one hundred years after Du Bois’s scholarship, racial health inequities remain a central challenge for public health, and measurement of the primary contributing factor remains elusive. To date, researchers have primarily relied on self-reported exposures of racism, which are useful but have limitations.^14^ A 2018 study found just twenty articles published between 2007 and 2017 that measured structural racism.^10^ Further, in 2021 Nancy Krieger and colleagues found that although the top four medical journals saw a dramatic increase in the number of articles that included the word *racism* in 2020, fewer than 10 percent of the sixty-four articles in their study contained any measures of structural (or other forms of) racism.^15^

The development of sound measures of structural racism is an urgent public health issue. Research must go beyond documenting racial inequities in health, beyond exclusively focusing on the roles of individual-level health risks and resources, and beyond merely conceptualizing racism as a fundamental cause to quantifying structural racism and its insidious effects on health.^9^

In this article we extend prior research calling for rigorous empirical studies on the links between racism and health^9^ by outlining specific methodological approaches that will move the field forward. First, we highlight how the history of racism in the US affects the ways the nation should approach measurement of structural racism and its effects on health. Second, we highlight the role that geographical context plays in shaping the measurement of structural racism and the importance of aligning theories and proposed mechanisms with the geographic locations and units examined. Finally, we propose promising directions for future research that incorporate innovative methodological approaches for quantitative and qualitative measurement of the multifaceted nature of structural racism, its intersections with other systems of oppression, and its impact on public health.

## Historical Context

Historical context is critical for accurately measuring structural racism for rigorous antiracist health policy research. Structural racism has been a core strand in the fabric of US society since its inception, beginning with the genocide and colonization of American Indians and constitutional protections for the institution of slavery.^16^ Throughout history, the US has been a racialized society, characterized by the formation and reformation of socially constructed hierarchies of racial groups as well as by structural racism in political, social, economic, judicial, residential, and health care contexts, which undergird racial inequalities in nearly every facet of life.^12,17,18^ Despite popular narratives about racial progress and a “postracial” America, data suggest that structural racism is alive and well in the US.^19,20^ Theoretical and empirical research show that there has been a qualitative shift in the nature of structural racism from predominant overt, de jure forms (such as colonization, slavery, lynchings, and Jim Crow laws) to more covert, de facto forms of racism (such as racialized mass incarceration, disenfranchisement, and residential segregation).^20,21^

The history of structural racism in the US has important implications for how the nation should approach measuring it and its effects on health. A burgeoning body of research has shown links between Black Americans’ contemporary health and historical forms of racism. For example, studies have shown that historical state and county variation in the enforcement (and abolition) of Jim Crow are predictive of Black mortality rates.^6,22^ However, there are major gaps in understanding of the pathways through which contemporary health outcomes are shaped by historical structural racism.

We have identified several promising avenues for future research to investigate how the historical context of structural racism affects present-day public health. First, to understand the broader impact of historical structural racism, future studies should examine how modern health is shaped by a wider array of past forms of structural racism, such as slavery, lynching, unequal treatment in the criminal-legal system, forced sterilization, and other manifestations of racialized violence.

Second, more empirical research is needed on the connections between historical and contemporary forms of structural racism. Theory suggests inextricable links, with historical forms directing, constructing, and molding contemporary structural racism.^21,23–25^ One empirical study showed that counties and states that had larger enslaved proportions of the population in 1860 have greater present-day inequalities in poverty and economic mobility and higher levels of pro-White bias.^25^ Another study showed that higher concentrations of slavery in 1860 at the county level are associated with slower declines in heart disease mortality among Blacks in recent decades, an association partially explained by intervening socioeconomic factors.^26^ Research has also shown that New Deal policies expanded the White middle class and are directly implicated in modern Black-White inequalities in wealth^27^ and that historical redlining practices underlie contemporary residential segregation patterns and health inequities.^28,29^

Third, future research should test how historical structural racism affects modern health outcomes either directly or indirectly via contemporary structural racism. Finally, further research is needed to identify the specific biopsychosocial mechanisms linking historical structural racism to health outcomes. There are important questions to be addressed: Does historical structural racism trigger a cascade of racialized social, political, and economic disadvantages that accumulate across the life course?^30^ Have social traumas of historical structural racism transmitted across generations via biological and psychosocial processes, and if so, how?^31^ What role do stress processes play in the embodiment of historical structural racism leading to health inequities?^4,32^

## Geographic Context

Structural racism manifests in different ways across geographic contexts. Researchers thus must determine how space and place and administrative, cultural, and physical boundaries operate and interact to produce and maintain these structures. Most important, researchers should ensure that the geographic units studied align with theory and research questions.

### MEASUREMENT OF STRUCTURAL RACISM AT THE STATE LEVEL

A large body of research has documented the important role of US state-level social, economic, and policy context in shaping the distribution of health and illness.^33,34^ This is because federalism, as defined in the Tenth Amendment of the United States Constitution, delegates to the states all powers not explicitly outlined or outlawed by the Constitution. Assertions of these state-level powers have ranged from the enslavement of Africans in the earliest points of US history to how education, housing, and infrastructure are financed and implemented. Since the 1980s the devolution of federal authority and preemption of local policy have increasingly consolidated power at the state level, making the states especially powerful influences on the social determinants of health in the US.^33^ State policies and practices shape employment, education, incarceration, real estate, taxes, health care, and many other factors affecting people’s lives. Many of these policies and practices are not race-neutral in their intentions, implementation, or effects.^35,36^ In fact, US states have a long history of contributing to racial oppression, from the de jure racism of the Jim Crow era to the contemporary de facto racism evident in policies and practices such as voter disenfranchisement and mandatory minimum sentencing.^23,24^ Thus, US states are best understood as racializing institutional actors shaping population health.^37^

An emerging line of research finds that state-level structural racism is associated with higher rates of infant mortality, myocardial infarction, functional limitations, depression, higher body mass index, and worse self-rated health among Black people.^37–39^ To date this research has focused on economic, social, and political contexts. More attention is needed in future research to examine specific policies and practices that create and exacerbate structural racism across a variety of domains. For example, many state and local jurisdictions rely on fines and fees to balance their budgets, effectively turning police into revenue generators through excessive traffic stops.^40^ This often has a disproportionate impact on Black communities, leading to negative social, economic, and health consequences. Philando Castile was pulled over by police forty-six times in Minnesota for minor violations and was issued more than $6,000 in fines. It was during the forty-seventh stop that a police officer took his life.^41^ This and other directions for future research are especially important given the increasing policy-making authority of states.

### MEASUREMENT OF STRUCTURAL RACISM IN NEIGHBORHOODS

The predominant geographic level used to theorize and conceptualize structural racism has been the neighborhood. This is in part a result of racial residential segregation and the institutional and individual practices and policies that create and maintain physical separation from White communities. Racial segregation is a fundamental cause of health disparities because it has been such an effective conduit of resources by state, federal, and even local governments.^42^ A recent systematic review found that segregation is the primary operationalization of structural racism in epidemiologic work,^10^ with a particular focus on existing patterns of segregation, historic redlining, or the development of contemporary discrimination indices. Although segregation alone, in addition to measures of redlining, has been shown to be predictive of racial health disparities, scholars have called for more nuanced measures of structural racism.^43,44^

Another reason the neighborhood has been a key geographic level for the measurement of structural racism is that existing neighborhood effects research provides both a theoretical and methodological framework that aligns well with the study of structural racism.^44^ Underlying neighborhood effects research is the theory that observed clustering of health behaviors and outcomes in hyperlocal settings is in part a result of the neighborhood context itself, rather than the individuals who live there. In the study of structural racism, the context is the specific political or institutional forces maintaining White supremacy. How the neighborhood is operationalized in research has varied greatly, including census tracts, ZIP codes, census-derived ZIP Code Tabulation Areas (ZCTAs), or even local administrative neighborhoods. Decisions on what constitutes a neighborhood are often determined by the availability of data rather than by how communities define themselves or what may be most relevant to answer the research question.

What areal unit is the most appropriate for structural racism measurement is an unsettled debate among scholars. Measuring structural racism at different geographic scales (that is, census tract, county, or state) causes the modifiable area unit problem,^45^ contributing to inconsistent findings concerning the health effect of structural racism. This problem arises because the boundaries designed to group populations are arbitrary—mere approximations of the demographics of the underlying population. As boundaries change, both the denominator and the numerators of what has been measured also change. A real-life example of the modifiable area unit problem is also another source of structural racism: Redistricting and gerrymandering are used to change political landscapes by physically changing which residents are within which boundaries.

For the impact of the modifiable area unit problem in structural racism and health research to be minimized, the areal unit for measurement must “make sense.”^44^ For example, to study the impact of racist policies and practices designed to keep Black people out of the so-called White neighborhood, scholars have used census block groups^46^ and ZCTAs^47^ as units of measurement. To examine the role of states as racialized agents, measurement at the state level is appropriate.^48^ For structural racism that operates at the labor market with no rigid boundaries, measurement using commuting zones has been proposed.^49^ To capture structural racism in densely populated counties that are urban and rural, measurement of structural racism using the Centers for Disease Control and Prevention’s Public Use Microdata Area, which account for geographic boundaries and population density, may allow scholars to capture the heterogeneity in structural racism across different pockets.^50^

Examples provided here are by no means a comprehensive list or suggestions of the “right” way to measure structural racism, nor are they an indication that the modifiable area unit problem can be completely avoided in structural racism research. Rather, we encourage the use of a theory-driven approach in which appropriate geographic units are selected on the basis of the proposed underlying mechanisms of structural racism, suggested by prior research and theory.

## Approaches To Capturing The Multifaceted Nature Of Structural Racism

Grounded in foundational scholarship on residential segregation as a driving force for health inequities,^1^ population health scholars use the ecological framework to guide how structural racism in other domains beyond housing access is measured. Of the many indices used, the index of concentration at the extremes^51^ and iterations of the index of disproportionality^39^ are most common. These indices operationalize structural racism as inequitable restriction of economic and sociopolitical resources (such as income, education, or the ability to vote) or disproportionate burden (such as police surveillance) experienced by members of racial and ethnic minority groups relative to White people.^10^ Measuring inequities ecologically instead of asking individuals to report their experiences with structural racism allows scholars to capture opaque aspects of structural racism to which minority people are exposed.^51^

### DATA SOURCES

One approach to expanding the measurement of structural racism is to seek out new data sources that capture understudied aspects of structural racism.^44^ To date, data used to measure structural racism often use population estimates from the Census Bureau’s decennial census and the intercensal American Community Survey. However, these publicly available data primarily focus on the composition of the geographic unit being analyzed rather than the context believed to be affecting residents.

One underused data source that can provide critical information for the study of structural racism is the Census Bureau’s Census of Governments. The Census of Governments collects financial information, including revenue, expenditures, debts, and assets, from all county, subcounty, and state governments in the US every five years, as required by federal law.^53^ These data provide information on how public money is spent and its flow across levels of government, providing insight on financial decision making by governmental entities. Patterns in education funding, police expenditures, cash assistance, and other pecuniary choices related to structural forces influencing the health and well-being of constituents can be abstracted from these data.

### MULTIFACETED NATURE OF STRUCTURAL RACISM

Gilbert Gee and Margaret Hicken posit that “racial inequities in…health will persist until we redirect our gaze away from specific institutions (and specific individuals), and instead focus on the resilient connections among institutions and their racialized rules.”^54^ Functioning similar to a system, various forms of structural racism share the same pathway (for example, education inequity leads to employment inequity) or interact with one another and exert both their independent and joint effects to cause poor health among members of racial and ethnic minority groups. As such, measures of structural racism should reflect this multidimensionality.

Measuring structural racism as a multifaceted determinant of health can be done in two ways. First, the approach taken by a majority of population health scholars is to measure various forms of structural racism as a set of exposures. In other words, a system is just the sum of its parts. For example, the pioneering work of Alicia Lukachko and colleagues measured inequities in political participation, employment, education, and judicial treatment and linked each form of structural racism to the risk for myocardial infarction.^39^ Yet the extent to which these forms of structural racism reinforce one another was not examined. Although findings from studies that use this approach to focus on one or a few forms of structural racism may provide focused policy recommendations, those recommendations may be incomplete or have unintended consequences because they do not consider how other dimensions of structural racism work behind the scenes to alter the effectiveness of policy changes.

Instead of examining forms of structural racism separately, new measurement approaches are being developed that capture the multifaceted nature of structural racism as a system. For example, three recent studies have proposed measuring structural racism as a multifaceted exposure using latent variable models.^37,50,55^ These latent variable approaches avoid measurement errors associated with observed variables, and they are well suited for measuring complex properties of structural racism. The approaches assume shared variance between structural racism indicators, allowing researchers to estimate an unbiased effect of a multifaceted system of structural racism on health.^56^ Although these methods are innovative, each has its pros and cons and may be applicable for different study designs and research questions.

### INTERSECTIONALITY

Another promising methodological consideration is incorporating intersectionality. The core insight of intersectionality is that individual life chances are shaped not by a single status hierarchy but by multiple overlapping systems of oppression such as racism, sexism, and classism.^48,49^ During the past decade intersectional studies have greatly advanced knowledge of health disparities, but little research has applied an intersectional lens to the study of structural racism and health. A straight-forward intersectional approach to structural racism and health research would involve investigating how structural racism exposure interacts with individual-level characteristics to shape health—for example, if gender or socioeconomic status moderates the impact of structural racism among Black people. In addition, scholars are beginning to develop novel structural intersectionality approaches.^48,50^ A structural intersectionality approach involves measuring structural racism and structural sexism^54^ and other systems of oppression in a particular social context to explore how they relate to one another to jointly shape population health, defined by specific constellations of individual-level statuses (for instance, middle-class Black women).^50^ Other approaches have created new intersectional measures such as structural gendered racism.^55^

### QUALITATIVE AND COMMUNITY-BASED PARTICIPATORY RESEARCH

Qualitative research also plays a critical role in the understanding of structural racism and its impact on health. Indeed, qualitative data provide rich information about the lived experience of structural racism by allowing people closest to the reality of structural racism to describe how racism affects their lives.^57–59^

Effective policy and authentic antiracist research must be born from within the affected community and subsequently cultivated by the community. Efforts to create measures of structural racism should be informed by community input, including community-based participatory research^60^ and public health critical race praxis principles.^61^ In Minnesota, for example, community conversations were held in virtual settings to hear from people about how structural racism affects their lives, how it should be measured, and whether the right measures are being used currently.^62^ This community research also helped identify domains of structural racism that have not yet been captured quantitatively. In addition, Brittany Chambers and colleagues conducted qualitative work with Black women in California to conceptualize structural racism from the perspectives of Black women across the reproductive lifespan.^63^ Themes that emerged from this study both confirm and introduce new domains of structural racism that can inform measurement and policy recommendations to improve health outcomes.^63^ Incorporating community voices has the potential to deeply inform the development of sound structural racism measures.^64,65^

## Conclusion

The world must dismantle structural racism to achieve health equity. Valid, replicable, and theoretically derived measures of structural racism are urgently needed to build evidence of its harms to population health and to identify pathways for intervention to advance racial health equity. Measuring structural racism for antiracist health policy research is both critical and urgently needed. In February 2021 Rep. Ayanna Pressley (D-MA) introduced the Anti-Racism in Public Health Act, highlighting the need for “robust, comprehensive research on the public health impacts of structural racism and policy solutions to bring an end to these disparities once and for all.”^66^

In this article we have offered a few considerations that are critical for moving the measurement of structural racism forward. The principles and approaches we identified here should also be applied to parallel areas of struggle and activism, such as emerging efforts to measure structural xenophobia in the form of immigration policy.^67^ It is also important to examine structural racism on a global scale and scrutinize how the colonization of countries in the Global South by imperialist majority-White countries functions as an international form of structural racism that undermines the health of existing populations.^68^

More than thirty years ago James Baldwin asserted that the nation had run out of time to address racism. Now Americans are faced with even more urgency. We have highlighted methodological considerations that will move the field forward in its ability to validly measure structural racism for the purposes of achieving health equity. This work is urgently needed—we have run out of time.
